# *Notes from the Field:* An Outbreak of *Salmonella* Agbeni Infections Linked to Turtle Exposure — United States, 2017

**DOI:** 10.15585/mmwr.mm6748a5

**Published:** 2018-12-07

**Authors:** Lia Koski, Lauren Stevenson, Jasmine Huffman, Amy Robbins, Julia Latash, Enoma Omoregie, Kelly Kline, Megin Nichols

**Affiliations:** ^1^Division of Foodborne, Waterborne, and Environmental Diseases, National Center for Emerging and Zoonotic Infectious Diseases, CDC; ^2^Oak Ridge Institute for Science and Education, Oak Ridge, Tennessee; ^3^CAITTA, Inc., Herndon, Virginia; ^4^New York State Department of Health; ^5^New York City Department of Health and Mental Hygiene; ^6^CDC/CSTE Applied Epidemiology Fellowship; ^7^Pennsylvania Department of Health.

In June 2017, PulseNet, the national molecular subtyping network for foodborne disease surveillance, identified 17 *Salmonella* Agbeni clinical isolates with indistinguishable *Xba*I enzyme pattern (outbreak strain) by pulsed-field gel electrophoresis. The same *Salmonella* Agbeni *Xba*I pattern was isolated from a turtle in 2015; in a 2016 investigation involving the same outbreak strain, 63% of patients reported contact with turtles (CDC, unpublished data, 2016). Despite prohibition of sale of small turtles (shell length less <4 inches) in the United States since 1975 (*1*), illness outbreaks associated with turtle contact continue to occur. Ill persons in previous *Salmonella* Poona and *Salmonella* Pomona outbreaks linked to turtles were geographically concentrated in the Southwest region of the United States (*2*,*3*). Turtle production is known to be higher in the Southeast region of the country (*2*). An outbreak investigation by CDC and health departments was initiated to identify the source of the 2017 illness outbreak.

A case was defined as isolation of *Salmonella* Agbeni with the outbreak strain from an ill patient during April–December 2017. State and local health officials interviewed patients to ascertain turtle exposure information, including details about the species of turtle and purchasing information. Purchase locations reported by patients were contacted for traceback information. Whole genome sequencing (WGS), using high quality single nucleotide polymorphism (hqSNP) analysis, was performed by CDC on clinical isolates from the 2017 outbreak, the 2016 illness cluster, and the turtle isolate from 2015 to characterize genetic relatedness.

Seventy-six cases were identified in 19 states in 2017; two thirds (67%) of patients resided in East Coast states (Connecticut, Delaware, Maryland, Massachusetts, New Jersey, New York, Pennsylvania, Rhode Island, and Virginia).* Patient ages ranged from <1–100 years (median = 21 years). Among 63 (83%) patients with information on hospitalization, 30 (48%) were hospitalized; no deaths were reported. Fifty-nine (78%) patients provided exposure information, including 23 (39%) who reported contact with turtles; among these, 14 (61%) specified small turtles. Among 12 patients who reported how the turtles were obtained, six purchased them from a street or roadside vendor, three purchased them from a retail store, two purchased them at festivals, and one reported receiving them as a gift. The traceback investigation did not identify a common turtle farm that supplied purchase locations. WGS hqSNP analysis indicated that the 2017 and 2016 clinical isolates and the 2015 turtle isolate were closely related, differing by 0–18 SNPs.

This salmonellosis outbreak was linked to contact with small turtles and was associated with a higher frequency of hospitalization (48%) than multistate foodborne pathogen outbreaks (27%) as well as recent *Salmonella* outbreaks linked to turtles (28%–33%) (*2*–*4*). The geographic distribution of patients differed from that of previous outbreaks, suggesting the need to better understand the breeding of turtles and distribution of turtle sales in the United States. WGS hqSNP analysis was used to link historic illnesses and turtle isolates to isolates from 2017 patients, supporting the hypothesis that turtles were the likely source of this outbreak. This outbreak indicates further need to educate consumers and retail store staff members regarding the ban on sale of small turtles and to educate consumers to prevent transmission of *Salmonella* from pets to humans.

## Footnotes

* https://www.cdc.gov/salmonella/agbeni-08-17/map.html.
